# Osteochondrolipoma of the Mandible

**Published:** 2017-12-01

**Authors:** Takeshi Kitazawa, Masato Shiba

**Affiliations:** Department of Plastic and Reconstructive Surgery, Matsunami General Hospital, Gifu, Japan

**Keywords:** mandible, osseous and cartilaginous metaplasia, osteochondrolipoma, mesenchymoma, pluripotency

## Abstract

**Objective:** Lipomas are very common benign tumors located in any part of the body in which fat is normally present, but lipomas containing both osseous and cartilaginous elements are rare. **Methods:** A case of osteochondrolipoma in a 72-year-old man is reported. The tumor in the mental region was 2×1.5×1.5 cm. **Results:** After resection of the tumor, there has been no recurrence during the 6-month postoperative follow-up. Histological examination confirmed the definitive diagnosis. **Conclusions:** Osteochondrolipoma is an extremely unusual lesion that should be kept in mind in the differential diagnosis of soft-tissue tumors.

Lipomas are common benign soft-tissue tumors that are composed of mature adipose cells, with uniform nuclei identical to the cells in normal adult fat and cause few clinical problems. Lipomas occasionally contain other mesenchymal elements such as blood vessels, fibrous tissue, and, less frequently, bone, or cartilage.^1^ An extremely rare case of lipoma at the mentum with concurrent osseous and cartilaginous differentiation is described.

## CASE REPORT

**Figure 1:**
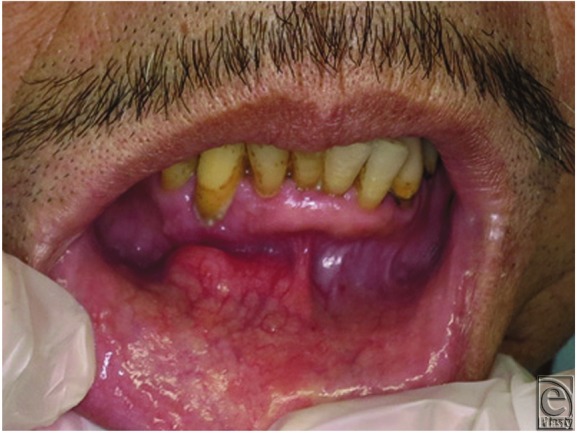
Clinical view of the lesion at first presentation.

A 72-year-old man presented with a 20-year history of a slow-growing painless mass at the mental region. On examination, there was a single, well-defined, mobile, relatively hard mass, with normal surrounding skin and mucosa (Fig 1).

**Figure 2:**
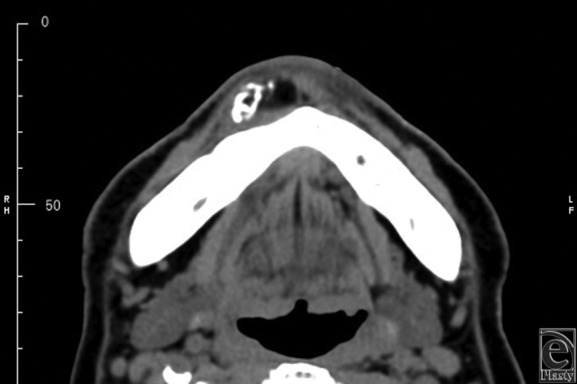
Computed tomographic scan shows a smooth mass of partial calcification at the mental region.

Computed tomographic scan showed an elliptically shaped smooth mass of fat density measuring 2×1.5 cm with partial ossification. The mass was not in contact with the mandible, and a clinical diagnosis of lipoma with calculus was made (Fig 2).

**Figure 3:**
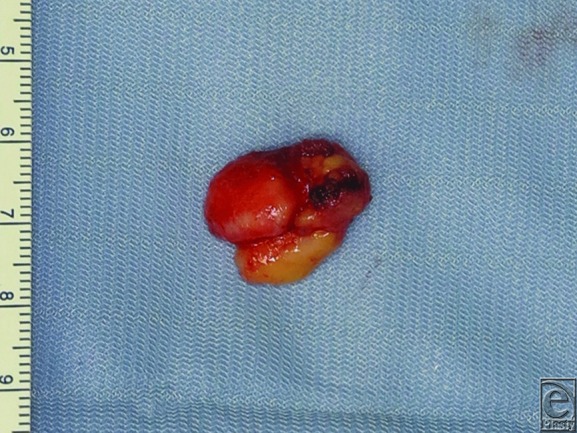
Macroscopic findings of the lesion. A soft yellowish mass together with a hard whitish nodule is seen.

**Figure 4:**
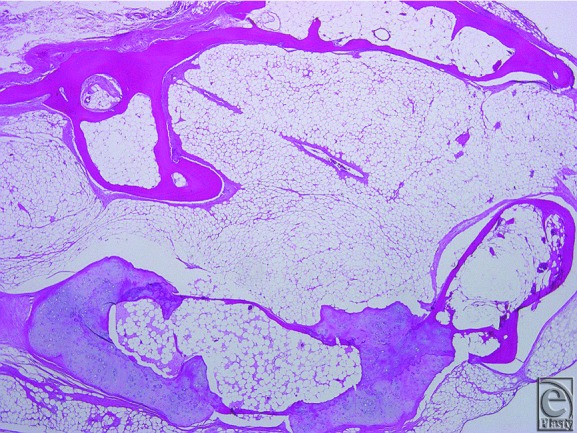

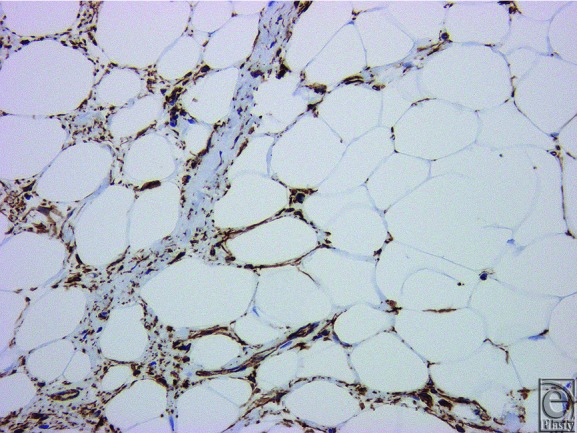
Histological and immunohistochemical findings of the specimen. (a) Predominant adipose tissue with osseous and cartilaginous differentiation (H&E, ×40). (b) Immunohistochemical stain showing positivity with CD34 (×100).

Under local anesthesia, the tumor with no adhesions to the periosteum of the mandible was easily dissected through the intraoral incision. Gross examination showed a whitish, partially yellowish mass measuring 2×1.5×1.5 cm, with a well-circumscribed smooth surface (Fig 3). Histological examination showed that the tumor was mostly composed of mature adipose tissue with partially osseous and cartilaginous differentiation. No atypical cells or mitotic figures were observed. Immunohistochemical staining of the tumor was positive for CD34. On the basis of these findings, the tumor was diagnosed as an osteochondrolipoma (Fig 4). Six months after surgery, there was no recurrence of the disease.

## DISCUSSION

Lipomas are very common benign soft-tissue tumors that are normally found at the back, neck, shoulders, abdomen, or proximal extremities.^1,2^ They occasionally contain other mesenchymal components such as bone, cartilage, and vessels, and they are called osteolipoma, chondrolipoma, and angiolipoma, respectively. Among them, osteolipoma and chondrolipoma are rare, especially lipoma with both osseous and cartilaginous components. That is, osteochondrolipoma is extremely rare and to the best of our knowledge, only 14 cases have been reported in the English literature, including the present case (Table 1); 5 tumors were localized at the maxillofacial region,^5^^-^8 with another 5 cases at the extremities,^2^^,^^4^^,^^9^^,^^12^^,^^13^ and the other 4 at the trunk.^1^^,^^3^^,^^10^^,^^11^ There was no clear sex predominance (8 men, 6 women), and the average patient age was 57.4 years (SD = 13.5 years; range, 19-73 years). The clinical history is usually that of a painless, slow-growing mass, sometimes measured in years (mean >5 years, ranging from 1 month to >20 years). Average tumor size was 4.2 cm (SD = 2.5 cm; range, 0.5-9.5 cm).

The nomenclature of osteochondrolipoma is controversial. Jones et al^14^ defined a tumor composed of 2 or more mature mesenchymal tissues, with no single element predominating, as mesenchymoma. In contrast, because the reported tumors including the present case had mature fatty tissue as the predominant component, it is appropriate to consider them osteochondrolipomas. However, there is no clear border between benign mesenchymomas and osteochondrolipomas.^3^

The pathogenesis of osteochondrolipomas remains uncertain. Different theories have been proposed to explain the formation of cartilaginous and osseous tissues in lipomas.^6^ One theory suggests that adipose, cartilaginous, and osseous components originate from multipotent undifferentiated mesenchymal cells independently.^15^ A second theory suggests that cartilaginous and osseous components may represent a metaplastic process in preexisting lipoma^3^^,^^16^ or chondrolipoma.^12^ Katzer^3^ suggested that, because the adipose tissue predominates in chondrolipomas and osteolipomas, and one can see different stages of the formation of cartilage and bone simultaneously, the pathogenesis of chondrolipomas and osteolipomas as primary mixed tumors is improbable. Recently, it has been shown that pluripotent adult stem cells, the adipose-derived stem cells, obtained from liposuction waste have the potential for chondrogenesis, osteogenesis, and myogenesis.^17^^,^^18^ As in the present case, CD34 was positive and pluripotency of the tumor was suggested. Considering that lipoma cells might have the potential to differentiate into other mesenchymal tissues, it would be surprising that most lipomas are pure lipomas without showing multidirectional differentiation.

The differential diagnosis should include osteocartilaginous choristoma, metastatic chondrosarcoma or osteosarcoma, liposarcoma with metaplasia, and posttraumatic chondrification.^19^ Because an incisional biopsy, which samples only a part of the lesion, can lead to misdiagnosis due to the pleomorphism of osteochondrolipoma, submission of the whole tumor for histopathological examination following exenteration is the preferred approach to diagnosis and treatment. No recurrences have been reported.

## Figures and Tables

**Table 1:** Previously reported cases of osteochondrolipomas

No.	Authors	Age, y	Sex	Location	Periosteal adhesion	Size, cm	Duration
1	Katzer^3^	55	F	Ischial region	Not mentioned	9.5×7×4.5	…
2		19	F	Left forearm	+	1.9	…
3		41	M	Left groin	−	8×5×4	…
4	Rau et al^4^	70	M	Left femur	−	8	…
5	Kuyama et al^5^	59	M	Lower lip	Not mentioned	0.9×0.5×0.5	2 mo
6	Tasić et al^6^	60	F	Tongue	−	2.0×1.7	5 y
7	Soulard et al^7^	61	M	Submandibular region	+	4.5×4.5×4	>20 y
8	Gültekin et al^8^	64	M	Mandibular symphysis region	+	2	2 mo
9	Ensat et al^9^	73	M	Left palm	−	6.5×6×4.5	5 y
10	Sunohara et al^10^	59	F	Left axilla	+	7.9×7.6×9.0	5 y
11	Nisio et al^11^	49	M	Left scapular region	−	3.0×3.0	1 mo
12	Tomonaga and Kudawara^12^	58	F	Left thigh	−	3×4	3 y
13	Choi et al^13^	63	F	Left popliteal fossa	−	4×5×3	>1 y
14	This study	72	M	Mandibular symphysis region	−	2×1.5×1.5	>20 y
